# Human Pulmonary Dirofilariasis, North Queensland, Australia, 2023^1^

**DOI:** 10.3201/eid3207.260280

**Published:** 2026-07

**Authors:** Kimberley Murray, Emily Grahn, Andrew Stacey, Carly Hughes, Harsha Sheorey, Constantin Constantinoiu, Leslie Kuma, Richard S. Bradbury

**Affiliations:** Townsville University Hospital, Townsville, Queensland, Australia (K. Murray, A. Stacey, C. Hughes, L. Kuma); James Cook University, Townsville (E. Grahn, C. Hughes, C. Constantinoiu, R.S. Bradbury); St Vincent’s Hospital Melbourne, Melbourne, Victoria, Australia (H. Sheorey); Royal Melbourne Hospital, Melbourne (H. Sheorey).

**Keywords:** *Dirofilaria immitis*, human pulmonary dirofilariasis, pulmonary adenocarcinoma, zoonoses, parasites, parasitic infection, heartworm, vector-borne infections, mosquito-borne, vector-borne disease, lung cancer, Australia

## Abstract

*Dirofilaria* nematodes, a common cause of canine filarial disease, are increasingly recognized as emerging human pathogens. We report a case of human pulmonary dirofilariasis in the lung of a man from Northern Australia with pulmonary adenocarcinoma. This case highlights the risk for zoonotic transmission in regions with high canine heartworm prevalence.

*Dirofilaria immitis* is a mosquitoborne filarial nematode that causes canine filarial disease. Although this parasite primarily affects canids, human dirofilariasis caused by several canine *Dirofilaria* spp. nematodes are increasingly being reported, especially in Europe and Asia (*1**-**4*). Humans are accidental hosts for *D. immitis* nematodes and become infected after the bite of a mosquito carrying *D. immitis* larvae. Larvae migrate through the circulatory system and die within the pulmonary vasculature, where they infarct small vessels, leading to a surrounding pulmonary granuloma (*5*). Those granulomatous nodules are often diagnosed incidentally on routine chest radiography and appear as single or multiple 0.5–4.5 cm round, dense, and opaque coin lesions in the lungs, which can be mistaken for primary or metastatic pulmonary malignancy (*1*,*5*). 

Human pulmonary dirofilariasis (HPD) caused by *D. immitis* infection is typically asymptomatic and self-limiting, and specific treatment is generally not necessary (*1*). Most cases of HPD are asymptomatic; wheezing, cough, hemoptysis, fever, chest pain, arthralgia, and malaise can develop (*1*). HPD is rarely reported (*2*,*3*), possibly underdiagnosed (*3*), and commonly misdiagnosed (*1*,*3*). We describe a case of HPD caused by *D. immitis* infection, identified incidentally in conjunction with primary pulmonary adenocarcinoma.

The male patient was 75 years of age and living in the tropical city of Townsville, Queensland, Australia; he was seen at a trauma visit in 2023. He reported a 100 pack/year smoking history, an occupational exposure to asbestos and silica, and a chronic and nonproductive cough. During his visit, imaging revealed a spiculated mass lesion measuring 35 × 28 mm in the right upper lobe that obstructed the posterior segmental bronchus and was closely associated with a separate nodule. 

The patient underwent a right upper lobectomy and mediastinal lymph node sampling for suspected primary pulmonary malignancy. Histopathologic and immunohistochemical evaluation of the pulmonary nodule confirmed a 34-mm solid-predominant primary adenocarcinoma. Gross dissection of the specimen revealed an additional nodule (Figure 1). Initial findings suggested multifocal disease; microscopy of the sample revealed a helminthic co-infection. We sought consultation for helminth characterization. The morphology of the worm within the second granulomatous nodule was most consistent with *D. immitis* (Figure 2). No further intervention was required for the *Dirofilaria* infection, although the patient continued management of the lung carcinoma.

**Figure 1:**
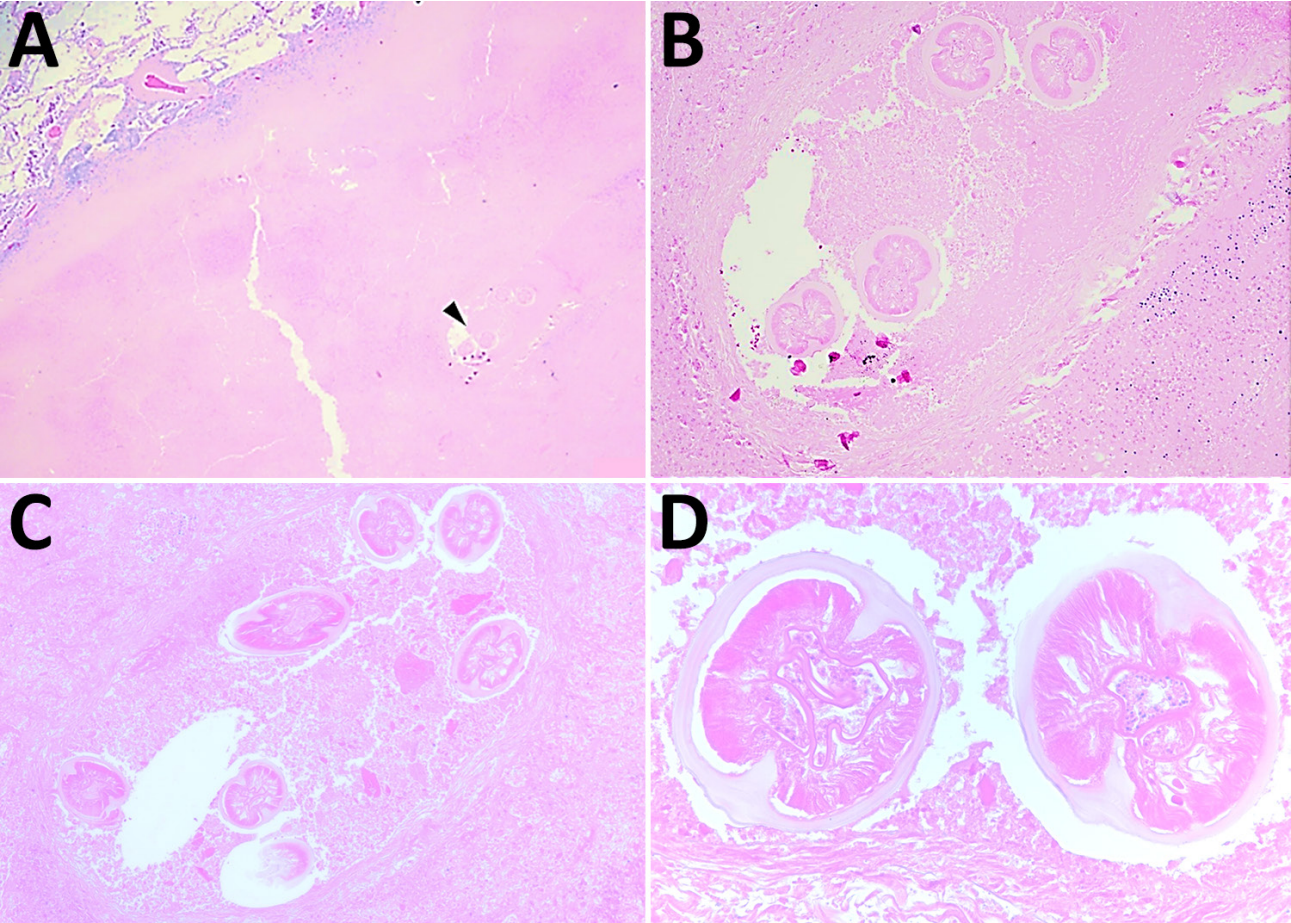
*Dirofilaria* organisms recovered from a patient with human pulmonary dirofilariasis in  North Queensland, Australia, 2023. A) Degenerate *D. immitis* nematode (black arrow) within a necrotic human lung granuloma and adjacent parenchyma. Original magnification ×2. B, C) Deeper cuts of the same region at 100× magnification. D) Cross-section of 2 regions of the coiled worm at 400× magnification. Hematoxylin and eosin stains.

**Figure 2:**
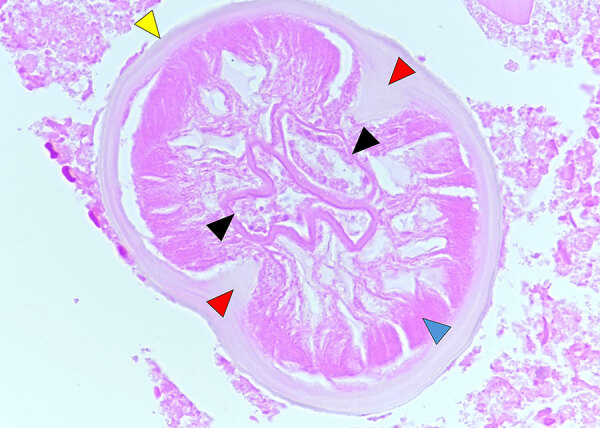
Defining anatomic features of *Dirofilaria immitis* within a small blood vessel (arteriole) in a necrotic human lung granuloma, recovered from a patient with human pulmonary dirofilariasis in Queensland, Australia, 2023. Yellow arrowhead indicates inflated necrotic smooth cuticle, without cuticular ridges; blue arrowhead indicates degenerate coelomyarian muscle structure; red arrows indicate inflated and necrotic internal cuticular ridges; and black arrows indicate degenerate paired uterine tubes. Hematoxylin and eosin stain; original magnification is ×600.

Identification of the helminthic parasite relied on characteristic morphologic features because DNA extraction and sequencing from the paraffin embedded specimen was not possible. However, *D. immitis* is the only canine *Dirofilaria* species known to occur in Australia (*6*,*7*). *D. roemeri*, a parasite of kangaroos and wallabies, is found in Queensland but is morphologically distinct in histological cross-sections (*5*,*7*).

Human infection remains rare in Australia; only 19 cases of *D. immitis*–related HPD were reported through 2012 (*3*), and only 1 additional case has been published since, also from North Queensland (*8*). A recent serosurvey of shelter dogs in Townsville revealed a high prevalence of *D. immitis* infection (<32%) (*9*), which could lead to increased zoonotic transmission.

*D*. *immitis* nematodes infect not only domestic dogs and cats but also wild canids (*2*). The widespread prevalence of heartworm in domestic dogs in Townsville might be attributable to dingoes (wild dogs of Australia) being common in bushland on the urban fringe of the city. Dingoes likely act as a sylvatic reservoir for infection of domestic dogs in Townsville (*9*). Previous necropsy surveillance studies of dingoes from the Townsville area found a heartworm prevalence of 75% (*9*).

We describe a case of *D. immitis* HPD in conjunction with primary adenocarcinoma. Similar coincidental findings of *D. immitis* infection and concurrent lung cancer have been previously reported in Texas, USA (*6*). Although those diagnoses were incidental, the overlapping clinical and radiologic features of lung cancer and pulmonary dirofilariasis pose a diagnostic challenge for clinicians and radiologists. Our report highlights the importance of preresection biopsy, meticulous gross dissection, and histologic sampling of the resection specimen for accurate diagnosis. Without those steps, the entire necrotic mass could have been included in the tumor measurement, potentially altering the tumor-nodes-metastasis stage and the associated prognosis. 

Clinicians, radiologists, and pathologists practicing in regions where canine heartworm is endemic should consider HPD in the differential diagnosis of pulmonary nodules. This case adds to the limited literature describing HPD caused by *D. immitis* nematodes in Australia and highlights the value of a One Health approach when evaluating emerging zoonotic infections in an endemic setting.
